# Is Heart Rate a Confounding Factor for Photoplethysmography Markers? A Systematic Review

**DOI:** 10.3390/ijerph17072591

**Published:** 2020-04-10

**Authors:** Md Rizman Md Lazin Md Lazim, Amilia Aminuddin, Kalaivani Chellappan, Azizah Ugusman, Adila A Hamid, Wan Amir Nizam Wan Ahmad, Mohd Shawal Faizal Mohamad

**Affiliations:** 1 Department of Physiology, School of Medical Sciences, Health Campus, Universiti Sains Malaysia, Kubang Kerian 16150, Kelantan, Malaysia; mdrizman@usm.my; 2 Department of Physiology, Faculty of Medicine, Universiti Kebangsaan Malaysia Medical Center, Jalan Yaacob Latif, Bandar Tun Razak, Cheras 56000, Kuala Lumpur, Malaysia; amyyra1234@yahoo.com.my (A.A.); dr.azizah@yahoo.com (A.U.); adilahamid@gmail.com (A.A.H.); 3 Centre of Advance Electronic & Communication Engineering (PAKET), Universiti Kebangsaan Malaysia, Bangi 43600, Selangor, Malaysia; 4 Biomedicine Programme, School of Health Sciences, Health Campus, Universiti Sains Malaysia, Kubang Kerian 16150, Kelantan, Malaysia; wanamir@usm.my; 5 Department of Medicine, Faculty of Medicine, Universiti Kebangsaan Malaysia Medical Center, Jalan Yaacob Latif, Bandar Tun Razak, Cheras 56000, Kuala Lumpur, Malaysia; superwall81@hotmail.com

**Keywords:** heart rate, cardiovascular disease, photoplethysmography, stiffness index, reflection index, second derivative of photoplethysmography

## Abstract

Finger photoplethysmography (PPG) waveform is blood volume change of finger microcirculation that reflects vascular function. Reflection index (RI), stiffness index (SI) and second derivative of photoplethysmogram (SDPPG) are derived from PPG waveforms proposed as cardiovascular disease (CVD) markers. Heart rate (HR) is a known factor that affects vascular function. Individual resting HR variation may affect RI, SI and SDPPG. This review aims to identify studies about the relationship between HR with RI, SI and SDPPG among humans. A literature search was conducted in Medline via the Ebscohost and Scopus databases to find relevant articles published within 11 years. The main inclusion criteria were articles in the English language that discuss the relationship between HR with RI, SI and SDPPG using PPG among humans. The search found 1960 relevant articles but only six articles that met the inclusion criteria. SI and RI showed an association with HR. SDPPG (SDPPG-b/SDPPG-a ratio, SDPPG-d/SDPPG-a ratio, aging index (AGI) and revised aging index (RAGI)) also had an association with HR. Only RI had a considerable association with HR, the association between SI and HR was non-considerable and the association between HR and SDPPG was inconclusive. Further interventional studies should be conducted to investigate this issue, as a variation in resting HR may challenge the validity of PPG-based CVD markers.

## 1. Introduction

Approximately more than 360,000 people died in 2016 due to coronary heart disease (CHD), the most common type of HD (heart disease), and CHD contributed to 43.2% of deaths in the United States [1] and was the top leading cause of death in 2016 in the United States [1]. Despite that, the cardiovascular disease (CVD) mortality trends for males and females in the United States declined from 1979 to 2016 [1]. In 2017, the main cause of death in Malaysia was ischemic heart disease, representing a percentage of 13.9% [2]. Death due to ischemic heart disease has increased by 54% compared with 10 years ago [2]. Ischemic heart disease was also the principal cause of death in urban areas (14.4%), while in the rural areas, pneumonia was the major cause of death at 13.4% [2]. The most common cause of death for the population aged 41–59 years in 2017 was ischemic heart disease at 17.8%, which increased by 0.8% from 2016 [2].

The continuous increase of baseline heart rate (HR) is also associated with a higher risk of CVD [3]. High resting heart rate (RHR) causes an increase in hemodynamic stress and a reduction of the diastolic time intervals, leading to increased oxygen consumption, reduction of myocardial perfusion and increased left ventricular (LV) workload in the long duration. These result in left ventricular hypertrophy (LVH), vascular stiffness, arterial hypertension, endothelial damage, coronary atherosclerosis, myocardial ischemia, ventricular arrhythmias and congestive heart failure (CHF) [4,5,6,7,8,9,10]. High RHR stimulates fatigue and fracture of elastic fibers inside the arterial wall [11]. Aorta stiffness further contributes to increased pulse wave velocity (PWV) and reflected wave velocity that lead to systolic hypertension, reduction of myocardial blood flow and organ impairment, which lead to increase cardiovascular morbidity and mortality [5]. In addition, high RHR is also a marker for sympathetic overactivity related to an increase of cardiovascular events risk [7,12,13,14,15]. Sympathetic overactivity increases the risk of obesity and metabolic syndrome that could stimulate insulin resistance, oxidative stress, inflammation, increased uric acid level, dyslipidemia and hypertension [5,9,16,17]. There are numerous biological mechanisms that show that sympathetic stimulation promotes both acute and chronic insulin resistance and may increase risk of diabetes: (1) sympathetic stimulation promotes vasoconstriction and reduces skeletal muscle blood flow that causes a reduction of glucose uptake into the skeletal muscle [18]; (2) sympathetic activation inhibits pancreatic b cells to secret insulin [19]; and (3) sympathetic overactivity induces the renin–angiotensin–aldosterone system that leads to increasing of HR and causes insulin resistance [20]. People with an increase in RHR between 50 and 60 years of age had poorer effect [21].

One method to assess CVD risk is by using a photoplethysmography (PPG) device [22]. A PPG device consists of a finger probe attached to an index finger and a laptop installed with a PPG calculation program. The finger probe contains two components, which include a light emitting diode (LED) (source of light) and a photodetector (light detector) [23]. Increased blood volume in the finger leads to less light detected by the photodetector, and reduced blood volume in the finger leads to more light detected by the photodetector. The changes of systoles and diastoles of the heart lead to changes of blood flow to the finger. Alternating changes of blood volume in the finger produce a pulse volume wave [24]. The PPG waveform displays the blood volume changes of the finger microcirculation that reflects the vascular function. The PPG concept has been used worldwide for assessing CVD risk. This method has potential for early screening of CVD risk among society members because of the mobility of the device and because it is non-invasive and an inexpensive method.

**Figure 1:**
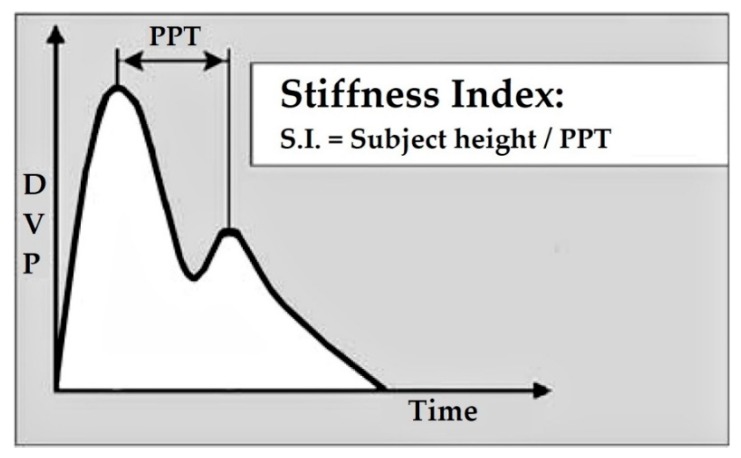
Stiffness index (SI) [27].

**Figure 2:**
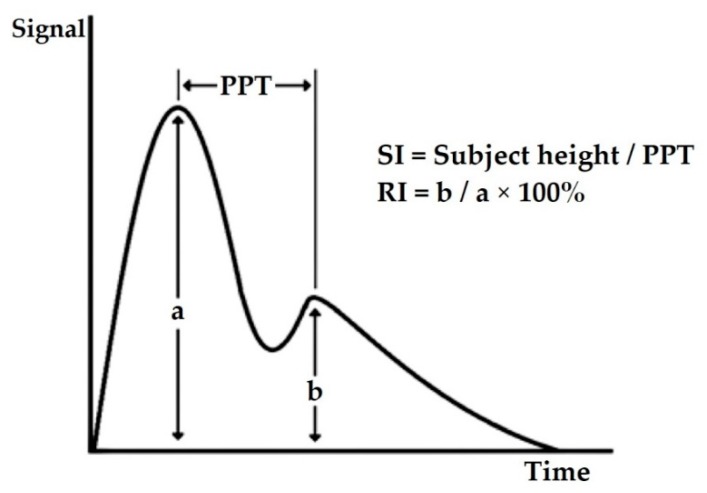
Reflection index (RI) [30].

Several vascular markers that have been derived from PPG waveform in assessing CVD risk are stiffness index (SI), reflection index (RI) and second derivative of PPG (SDPPG). SI determines the stiffness level of large arteries [25] and is associated with PWV in large arteries [26]. SI can be calculated by the formula: h/PPT [27,28] (Figure 1). h is the body height of the patient. The peak-to-peak time (PPT) is time between the early peak systolic peak (PPG-a) and the second diastole peak (PPG-b). RI measures the stiffness level of small to moderate arteries [29]. RI is calculated by the following formula: (PPG-b/PPG-a) × 100% [30] (Figure 2). RI is a ratio of diastole peak amplitude over the systole peak amplitude.

**Figure 3:**
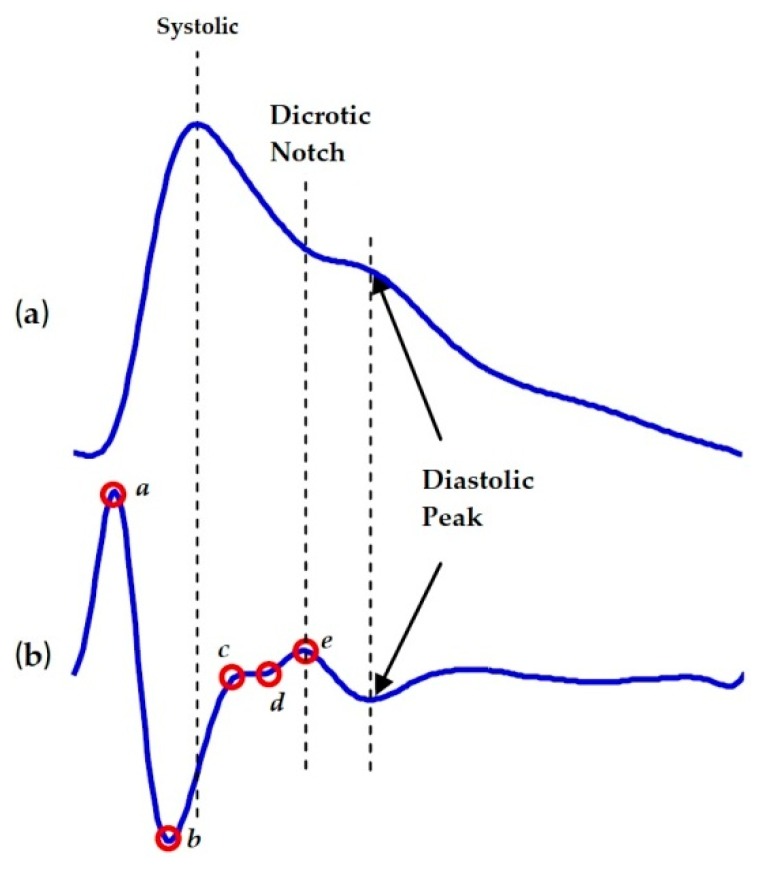
Second derivative of photoplethysmography (SDPPG) [39]. (**a**) Fingertip photoplethysmogram. (**b**) Second derivative wave of photoplethysmogram. The photoplethysmogram waveform consists of one systolic wave and one diastolic wave, while the second derivative photoplethysmogram waveform consists of four systolic waves (a, b, c, and d waves) and one diastolic wave (e wave).

The SDPPG consists of five waves, including SDPPG-a wave (early systolic positive wave), SDPPG-b wave (early systolic negative wave), SDPPG-c wave (late systolic re-increasing wave), SDPPG-d wave (late systolic re-decreasing wave) and SDPPG-e wave (early diastolic positive wave) [31] (Figure 3). The comparative heights of these waves (SDPPG-b/SDPPG-a, SDPPG-c/SDPPG-a, SDPPG-d/SDPPG-a and SDPPG-e/SDPPG-a ratios), mainly the SDPPG-b/SDPPG-a ratio, are associated with aging and carotid distensibility [32] and are used in computing the aging index [33]. A high SDPPG-b/SDPPG-a ratio indicates an increase in cardiovascular risk or atherosclerosis [34]. Takazawa et al. [35] revealed that the SDPPG-b/SDPPG-a ratio indicates raised arterial rigidity, and the SDPPG-b/SDPPG-a ratio rose with age. Imanaga et al. [32] found that the degree of the SDPPG-b/SDPPG-a ratio is associated with the distensibility of the peripheral artery and propose that it is a valuable non-invasive index of atherosclerosis and changes in arterial distensibility. Otsuka et al. [36] established that the SDPPG-b/SDPPG-a ratio is positively associated with the Framingham risk score. The Framingham risk score has been applied to approximate individual risk of cardiovascular heart disease. Their outcomes recommend that the SDPPG-b/SDPPG-a index might be useful to identify high-risk subjects for cardiovascular heart disease. The SDPPG-b/SDPPG-a ratio increased with an increment of age [34,37]. The SDPPG-d/SDPPG-a ratio demonstrates the intensity of reflecting pulse waves from peripheral arteries [34]. Takazawa et al. [35] revealed that the increasing SDPPG-d/SDPPG-a ratio represented a reduction of arterial stiffness; therefore, the SDPPG-d/SDPPG-a ratio decreased with increasing age and higher arterial stiffness [34,37]. Moreover, they established the (SDPPG-d/SDPPG-a) ratio as a valuable index for the assessment of vasoactive agents and as an index of left ventricular afterload. Aging index (AGI @ AI) is proposed to signify the distensibility of large arteries [35]. AGI demonstrates the overall vascular stiffness, and it increases by age [34]. AGI can be calculated by the following formula: [(SDPPG-b)-(SDPPG-c)-(SDPPG-d)-(SDPPG-e)]/(SDPPG-a) [34,35]. Revised aging index (RAGI) is clarified as a novel SDPPG index [38]. RAGI can be calculated by the following formula: [(SDPPG-b)-(SDPPG-d)-(SDPPG-e)]/(SDPPG-a) [38].

A previous study found that HR affects vascular markers, such as augmentation index (AIx) and PWV [40,41,42]. AIx represents systemic arterial stiffness, and PWV represents arterial stiffness [41,43,44]. The effects of HR towards PPG markers are still inconclusive. Because PPG markers had an association with arterial stiffness, it is speculated that HR may affect PPG markers [45]. The variation of the HR may influence the result of vascular markers and affect cardiovascular risk. Therefore, it is important to know the relationship between variations of HR with PPG markers in assessing the CVD risk. 

The objective of this review is to reveal the relationship between SI, RI and SDPPG with HR. The variation in HR among people may influence the result of the vascular markers measured and may question the validity of the test.

## 2. Materials and Methods 

A literature search was conducted to investigate the relationship between HR and SI and RI and SDPPG among human subjects. Medline via Ebscohost and Scopus databases were used to identify relevant articles published between 2009 and March 2019. Related articles were identified by using two groups of keywords: (1) heart rate* AND (2) reflection index* OR stiffness index* OR second derivative of finger photoplethysmography* OR finger photoplethysmography*.

The main inclusion criteria for the search results were limited to articles published in the English language with abstracts and full texts that discuss the relationship between HR, SI, RI and SDPPG, using PPG among human subjects. Studies using animal and tissue culture were excluded. In addition, review papers, letters, conference papers, articles in the press, notes, editorials and short surveys were excluded.

For the purpose of this review, only studies that showed a relationship between HR and SI, a relationship between HR and RI or a relationship between HR and SDPPG were selected.

Articles were filtered in three stages before being selected for the review. In the first stage, any article that did not meet the inclusion criteria based on the article title was removed. In the second stage, abstracts of the remaining articles were screened again, and papers that did not meet the selection criteria were discarded. Finally, the remaining articles were read carefully by two independent readers to remove articles that did not meet the selection criteria. All readers had to agree regarding the criteria of selected articles to be reviewed prior to the data extraction stage. Any conflicts of opinions between the reviewers were resolved through rationally mutual discussions. All data searching was done independently using a data search form. The following data were obtained from the articles: main author of the article; publication year of the article; population of subjects in the study; mean age of the subjects; age range among the subjects; percentage of male subjects; instruments used in the study; relationship between heart rate and the parameter; and significant level of the relationship.

**Figure 4:**
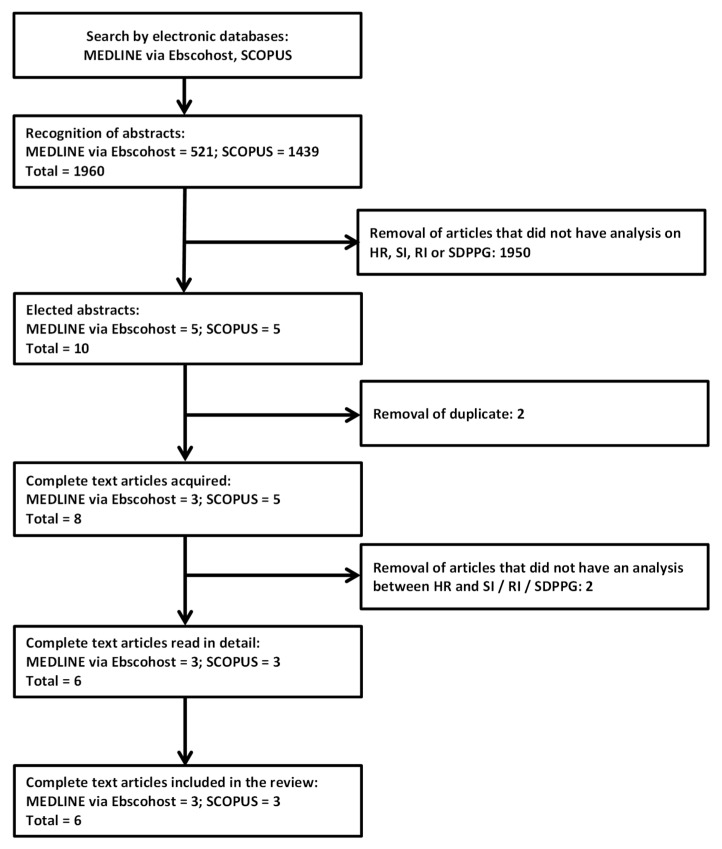
Flowchart of the election of the related articles.

The search found eight articles potentially related to the search criteria, of which six articles met the inclusion criteria (Figure 4).

## 3. Results

For the association analysis, several papers used simple correlation [33,34,46], while others used multiple regressions [38,47,48]. Multiple regression analysis provides stronger evidence, because other cofounder factors are included, such as enhancement of sympathetic activity [47], hypertension and hyperglycemia [38] and age and mean blood pressure [48].

For the association between SI and HR, out of four studies, only one study observed a significant negative association, but the association was poor (*r*^2^ = −0.06, *p* = 0.02) [48]. From four studies that showed an association between SI and HR, two studies used simple correlation [33,46], and another two studies used multiple regressions [47,48]. For the associations between RI and HR, out of three studies, only two studies observed significant negative associations, which were weak and moderate [(*r* = −0.4, *p* < 0.001) and (*r* = −0.35, *p* < 0.001)] [33,46]. From three studies that revealed an association between RI and HR, two studies used simple correlation [33,46], and another study used multiple regressions [47].

The secondary derivatives of PPG that were included were the b/a ratio, d/a ratio, AGI and RAGI. For the association between the b/a ratio and HR, out of two studies, only one study observed a significant negative association, but the association was poor (β = −0.200, *p* < 0.001) [38]. From two studies that showed an association between the b/a ratio and HR, one study used simple correlation [34], and another study used multiple regressions [38]. For the association between the d/a ratio and HR, out of two studies, only one study observed a significant positive association, but the association was also weak (β = 0.143, *p* < 0.001) [38]. From two studies that revealed an association between the d/a ratio and HR, one study used simple correlation [34], and another one study used multiple regressions [38]. For the association between aging index (AGI @ AI) and HR, out of two studies, only one study observed a significant negative association, but the association was poor (β = −0.057, *p* = 0.020) [38]. From two studies that showed an association between AGI @ AI and HR, one study used simple correlation [34], and another study used multiple regressions [38]. For the association between RAGI and HR, there was only one study that observed a significant negative association, but the association was poor (β = −0.192, *p* < 0.001) [38]. One study showed an association between RAGI and HR and used multiple regressions [38], and no study used simple correlation. Brief description of vascular markers is shown in Table 1.

The results of the related studies are in conflict with each other. The reasons for these findings include different types of study populations, different numbers of study subjects, different mean age of subjects and different age range of subjects involved in the studies. All this information is included in Table 2.

In general, SI showed a significant negative association with HR in one out of four studies. So, only 25% of studies showed an association between SI and HR. Because of that, the association between SI and HR was non-considerable. RI showed a significant negative association with HR in two out of three studies. So, 67% of studies showed an association between RI with HR. Because of that, in general, RI had a considerable negative association with HR. SDPPG showed a significant association with HR in one out of two studies. So, 50% of studies showed an association between SDPPG and HR. Because of that, in general, the association between SDPPG and HR was inconclusive.

## 4. Discussion

In this study, only RI had a considerable negative association with HR. The association between SI and HR was non-considerable, and the association between HR and SDPPG was inconclusive. Thus, it was suggested that HR may be a confounding factor for RI.

RI describes the magnitude of wave reflection from the lower limbs to the aorta [29]. RI is evaluated by using the formula (PPG-b/PPG-a) × 100% [30]. RI depends on blood volume changes in blood vessels, and blood volume, namely cardiac output/stroke volume, depends on the contraction of the heart. PPG is the method that evaluates changes of blood volume in blood vessels each time a heart beats [49]. From PPG waveform, RI measurement could reveal significant data regarding blood volume changes for high-risk subjects [49].

Contraction of the heart depends on the activity of the sinoatrial (SA) node. The SA node starts electrical impulses to induce contraction. The SA nodes are located in the atrial wall at the junction of the right atrium and the superior cava vein [50]. The blood volume ejected by the heart is correlated with the heart rate. Increasing the heart rate leads to increasing the cardiac output (CO) (CO = HR × SV). The amount of blood pumped out by the heart within 1 min is the cardiac output and stated as liters/minute. The HR, contractility, preload and afterload may influence the CO [48]. Understandably, the product of the stroke volume (SV) and the number of heart beats per minute (HR) is equivalent to CO [51].

Increased heart muscle contractions also promote the increase of blood volume ejected by the left ventricle to the aorta. More blood ejected to the aorta produces more cardiac output. The more blood ejected from the heart to the peripheral, the more reflected waves from lower limbs to the aorta will be decreased due to RI being negatively correlated with HR.

Stiffness index (SI) is recognized as a standard marker for large arterial stiffness [25,29,52,53]. SI value is calculated as height/peak to peak time. SI is an evaluation of the diastolic time relative to the systolic time of PWV in large arteries (body height divided by time between diastolic peak and systolic peak) [54]. Therefore, SI is provisional to the blood flow within the time between two peaks, which are the systolic peak and diastolic peak. The duration is equal to the time between the opening of the aortic valve to the closure of the aortic valve.

**Figure 5:**
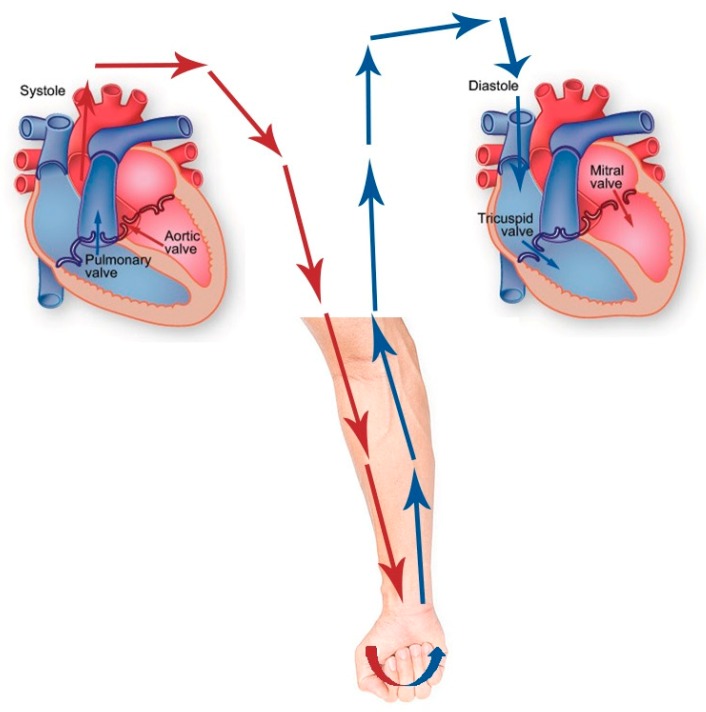
Blood flow from the aorta to the peripheral and back to the right atrium.

After performing exercise or any physical activities, HR will increase. A high HR leads to the reduction of stroke volume. In an acute condition after physical activities, there is no structural characteristic change in blood vessels, but a significant HR increase was recorded. This phenomenon explains that increased oxygen demand causes sympathetic stimulation, which leads to increased HR. So that the time duration of blood flow from the left ventricle via aortic valves to the aorta [55] and then to the peripheral [56] and finally back to the superior and inferior vena cava persists, flows into the right atrium, flows via tricuspid valves and flows into the right ventricle [57] (Figure 5) will not change, although the HR increased. Heart rhythm did not change, but heart rate increased after exercise or physical activities. Because of that, SI is not correlated with HR.

The increased SDPPG-b/SDPPG-a ratio demonstrates an increase in cardiovascular risk or atherosclerosis [34] and an increase in arterial stiffness and age [35]. The SDPPG-b/SDPPG-a ratio represents the blood flow from the opening of the bicuspid valve, flows into the left ventricle and flows via the opening of the aortic valve to the aorta. The blood flow is not influenced by HR. Therefore, the SDPPG-b/SDPPG-a ratio does not correlate with HR.

Takazawa et al. [35] discovered that the increasing SDPPG-d/SDPPG-a ratio characterized a reduction of arterial stiffness, so that the reduction of the SDPPG-d/SDPPG-a ratio represented increasing age and higher arterial stiffness [34,37]. The SDPPG-d/SDPPG-a ratio represented the blood flow in the aorta after the aortic valve opened. Exercise or physical activities may promote the increasing of HR that causes an increase of CO, leading to increased blood flow in the aorta. Vice versa, during resting conditions when HR is at a normal level, the presence of blood flow in the aorta justifies the correlation between the SDPPG-d/SDPPG-a ratio and HR.

### Study Limitations

This systematic review had a limited number of studies that had been included. This systematic review is also based on correlation analysis and did not include studies with pacemaker subjects or drug intervention.

## 5. Conclusions

In conclusion, HR may be a confounding factor for RI. However, this is based on a limited number of correlation studies, and further interventional studies using pacemakers or drugs should be conducted.

## Figures and Tables

**Table 1:** Brief description of vascular markers.

No.	Vascular Markers	Description
1.	Stiffness index (SI)	determines the stiffness level of large arteries [25]
2.	Reflection index (RI)	measures the stiffness level of small to moderate arteries [29]
3.	SDPPG-b/SDPPG-a ratio	high SDPPG-b/SDPPG-a ratio indicates an increase in cardiovascular risk or atherosclerosis [34]
4.	SDPPG-d/SDPPG-a ratio	increasing SDPPG-d/SDPPG-a ratio represented reduction of arterial stiffness [35]
5.	Aging index (AGI @ AI)	demonstrating the over-all vascular stiffness and it increases by age [34]
6.	Revised aging index (RAGI)	a novel SDPPG index [38]

**Table 2:** Result of systematic review.

Reference	Population (*n*)	Mean Age (Year) Median (Interquartile Range)	Age Range (Year)	Male (%)	Instrument	Statistical Analysis	Association between HR and PPG Vascular Markers		
							SI	RI	SDPPG
[33]	Type 1 diabetes mellitus (T1DM) (57) & healthy (53)	T1DM 32 (24–43) Healthy 27 (22–23)	not mentioned	43.6	PCA2 pulse contour analyzer (PulseTrace PCA2, Micro Medical)	Pearson’s correlation coefficient	*r* = 0.06, *p* = 0.64	*r* = −0.4, *p* < 0.001	not measured
[34]	Women undergoing in vitro fertilization (IVF) (68)	36 ± 5 36 (26–44)	Not mentioned	0	Meridian^TM^ DPA photoplethysmograph (Salcor AB)	Kendal tau rank correlation analysis	not measured	not measured	b/a (Tau = −, *p* = 0.70) (not significant) d/a (Tau = −, *p* = 0.79) (not significant) AI (Tau = −, *p* = 0.68) (not significant)
[38]	Healthy (1613)	65.3 ± 9.6	Not mentioned	39.6	Finger photoplethysmograms (Arteto; U-Medica)	Multiple regression analysis	not measured	not measured	b/a (β = −0.200, *p* < 0.001) d/a (β = 0.143, *p* < 0.001) AGI (β = −0.057, *p* = 0.020) RAGI (β = −0.192, *p* < 0.001)
[46]	Normal glucose tolerance (121), Impaired glucose tolerance (33) and type 2 diabetes mellitus (47)	Men (60.8 ± 5.2) Women (58.9 ± 5.7) Men (62.6 ± 3.5) Women (59.0 ± 5.1) Men (61.4 ± 4.9) Women (61.4 ± 3.1)	45–69	46.3	Finger photoplethysmography (PulseTrace, Micro Medical/Care Fusion)	Spearman rank correlations	*r* = 0.07, *p* > 0.05	*r* = −0.35, *p* < 0.001	not measured
[47]	Normal glucose tolerance or insulin resistance (26)	31 ± 10	18–60	38.5	Digital Photoplethysmograph (Pulse Trace System, Micro Medical)	Univariate regression analysis	β = −0.30, *p* = 0.132	β = −0.16, *p* = 0.438	not measured
[48]	Healthy (91)	54.1 ± 8.5	Not mentioned	38.5	Photoplethysmographic finger probe (PulseTrace 2000, Micro Medical)	Multiple linear regression analyses	*r*^2^ = −0.06, *p* = 0.02	not measured	not measured

Abbreviations: β: Beta (relationships of univariate regression analysis); T1DM: Type 1 diabetes mellitus; r: Correlation coefficient; AGI or AI: Aging index = [(SDPPG-b)-(SDPPG-c)-(SDPPG-d)-(SDPPG-e)]/(SDPPG-a); RAGI: Revised aging index = [(SDPPG-b)-(SDPPG-d)-(SDPPG-e)]/(SDPPG-a); IVF: In vitro fertilization; r2: Coefficient of determination/coefficient of multiple determination for multiple regression; Tau: Kendall’s rank correlation coefficient.
